# Validity and Reproducibility of a Food Frequency Questionnaire to Assess Nutrients Intake of Pregnant Women in the South-East of Spain

**DOI:** 10.3390/nu13093032

**Published:** 2021-08-30

**Authors:** Daniel Hinojosa-Nogueira, Desirée Romero-Molina, María José Giménez-Asensio, Beatriz Gonzalez-Alzaga, Inmaculada Lopéz-Flores, Silvia Pastoriza de la Cueva, José Ángel Rufián-Henares, Antonio F. Hernández, Marina Lacasaña

**Affiliations:** 1 Centro de Investigación Biomédica, Departamento de Nutrición y Bromatología, Instituto de Nutrición y Tecnología de los Alimentos, Universidad de Granada, 18071 Granada, Spain; dhinojosa@ugr.es (D.H.-N.); spdelacueva@ugr.es (S.P.d.l.C.); 2 Instituto de Investigación Biosanitaria ibs.GRANADA, Av. de las Fuerzas Armadas, 2, 18014 Granada, Spain; deromero@ugr.es (D.R.-M.); mariajoseases@hotmail.com (M.J.G.-A.); beatriz.gonzalez.easp@juntadeandalucia.es (B.G.-A.); ilopez@ugr.es (I.L.-F.); ajerez@ugr.es (A.F.H.); 3 Statistics and Operations Research Department, Faculty of Sciences, University of Granada, Fuente Nueva s/n, 18071 Granada, Spain; 4 Andalusian School of Public Health (EASP), 18011 Granada, Spain; 5 Department of Genetics, Faculty of Sciences, University of Granada, Fuente Nueva s/n, 18071 Granada, Spain; 6 Department of Legal Medicine and Toxicology, University of Granada School of Medicine, Health Sciences Technological Park, Avenida de la Investigación, 11, 18016 Granada, Spain; 7 CIBER Epidemiology and Public Health (CIBERESP), Instituto de Salud Carlos III, Monforte de Lemos 3-5, Pabellón 11, Planta 0, 28029 Madrid, Spain

**Keywords:** pregnant women, nutrition, intake, food frequency questionnaire, 24-h dietary recalls, Spain

## Abstract

Proper nutrition during pregnancy is pivotal to maintain good health for the child and the mother. This study evaluates the reproducibility and validity of a food frequency questionnaire (FFQ) designed to assess nutrient intake during pregnancy in the GENEIDA (Genetics, Early life Environmental Exposures and Infant Development in Andalusia) prospective birth cohort study. In addition, the nutrient intake was estimated and then compared with European guidelines and other studies. Diet information was collected from 690 pregnant women using a FFQ administered at two periods of pregnancy (used for the reproducibility study) and 24-h dietary recall (for the validity study). Statistical approaches included Spearman’s correlation coefficient and percentage agreement, classifying women into the same or adjacent quintiles to assess reproducibility, and limits of agreement (LoA) to evaluate validity. In the study of reproducibility, significant correlations for nutrients adjusted for total energy had an average of 0.417. Moreover, the percentage of subjects classified in the same quintile for nutrient intakes were above 66%. In the validation study, the significant correlation for nutrients adjusted for total energy had an average of 0.272. Nevertheless, the percentage of results in the LoA was above 94%. Our results were similar to other studies suggesting that the FFQ used is a valid tool of collect dietary intakes for South-East Spanish pregnant women.

## 1. Introduction

During pregnancy, women undergo physiological and anatomical changes [1], which modify nutritional requirements [2]. Several studies have identified that dietary changes during pregnancy could either improve or worsen newborn health. Moreover, maternal diet during pregnancy can affect the child’s development and growth [3]. Consequently, a healthy diet plays an important role in the fetal growth trajectories and the subsequent state of health [4,5]. One of the diets associated with better healthy eating habits is the Mediterranean diet, characterized by a high consumption of fresh fruit, vegetables, cereals, legumes, nuts, seeds, olive oil (main source of fats), moderate intake of fish, cheese, yogurt and only small amounts of red and processed meat [6]. The Mediterranean diet has been associated with lower risk of preterm birth [7], lower child adiposity [8], higher weight at birth [9], and lower offspring waist circumference at preschool age [10].

Despite Spain is located in the Mediterranean area, several epidemiological studies suggest that food patterns do not reach nutritional recommendations [11,12,13]. Accordingly, the evaluation of nutrients intake would result in a more accurate reflection of their adequacy with respect to recommendations [14]. However, valid instruments to assess dietary composition and nutrient intake during pregnancy are required [15]. Biochemical parameters are the most precise methods to study the nutritional status, but they only reflect nutritional status at a specific time and are also the most expensive approach [16,17]. Moreover, food records or 24-h dietary recalls may provide accurate information, although they are difficult to administer and a high level of cooperation is necessary [18]. The food frequency questionnaire (FFQ) is the most used method in epidemiological studies as it provides a better approximation of the usual dietary intake over a longer period [19]. FFQ is also a low-cost and easy-to-apply instrument [20] and thus it is one of the most used methods in pregnancy studies [21,22].

Dietary habits vary widely among the population according to different factors (e.g., geographic area, population type or cultural beliefs) and, therefore, the FFQ must be adapted and validated for use in each specific population [22]. Adaptations must take into account the type of food consumed, the accessibility to that food, the traditions or, in the case of pregnant women, the use of dietary supplements.

FFQs have to be validated, usually with 24-h dietary recalls [23,24,25] or biochemical parameters [26] in order to avoid errors and biases. The validity of the FFQ must be evaluated using several measures and statistical methods. The most commonly used method is the correlation coefficient, though this is not a measure of agreement but instead a measure of association that can be partly influenced by the size of the sample. The Bland-Altman method is also a good method to assess the level of agreement between different methods of measurement. Most epidemiological studies use more than one statistical approach to demonstrate the robustness of the validation and reproducibility process [27,28].

The aim of this study was to evaluate the reproducibility and validity of a FFQ designed to assess maternal nutrient intake during pregnancy in the GENEIDA (Genetics, Early life Environmental Exposures and Infant Development in Andalucia) project, a prospective birth cohort study of mother/child pairs conducted in the South-East of Spain, as well as to highlight the importance of the adequate intake of nutrients during pregnancy.

## 2. Materials and Methods

### 2.1. Study Design and Population

Participants involved in this study were healthy pregnant women from a population-based birth cohort study, the GENEIDA Project, set up in “El Poniente” district (province of Almeria, South-Eastern Spain) in April 2014. Eight hundred pregnant women in the first trimester of pregnancy (before 13 weeks of gestation) were enrolled in the study. They were followed-up during the second and third trimesters of pregnancy, delivery, and their children during the first 48 months of age.

Criteria for inclusion of the mothers were: (a) to be a resident of El Poniente district, (b) to be at least 16 years old, (c) to have a singleton pregnancy, (d) to not have followed any programme of assisted reproduction, (e) planning to deliver at El Poniente Hospital, (f) speak fluent Spanish, and (g) have no chronic disease diagnosed before pregnancy and not to be under medical treatment.

Of the 800 women initially enrolled in the study between April 2014 and November 2016, 690 women completed a FFQ at the first (weeks 10–13) and third trimesters of gestation (weeks 32–36). 24-h dietary recalls were completed by a subgroup of 43 women. This study was conducted according to the guidelines laid down in the Declaration of Helsinki and all procedures involving research and study protocol were approved by the Hospital Ethics Committee. Written informed consent was obtained from all participants or the legally responsible.

### 2.2. Data Collection

#### 2.2.1. General Questionnaire and Medical Records

Information relative to participants was obtained from a structured questionnaire administered by trained personnel in the first and third trimesters of pregnancy, and from medical records. This information consisted of socio-demographic characteristics (age, marital status, birth country, education level, household total net income), medical and reproductive history, self-reported pre-pregnancy weight and weight at 32 weeks of gestation, height, tobacco smoking and alcohol consumption, vitamin and mineral supplementation. Body mass index (BMI) was calculated by dividing pre-pregnancy weight (Kg) by the square of their height (meters). BMI was classified as underweight (<18.5), normal (18.5–24.9), overweight (25.0–29.9) and obesity (≥30) [3]. Smoking during pregnancy was defined as never, yes at the first trimester only, and yes during the entire pregnancy. Information on alcohol (g/day) intake was assessed in the FFQ with specific items for wine, beer, and liquor consumption, together with standard servings for all the items. The FFQ also collected information about vitamin and mineral supplement consumption.

#### 2.2.2. Dietary Assessment

A modified version of a previous validated semi-quantitative FFQ for Spanish population [18] was used. Additional food items (*n* = 40) were added in order to assess better the usual daily food intake of the Andalusian population.some food Thus, food groups such as legumes, cereals, oils, sausages, fish and industrial bakery products, among others, were disaggregated into individual categories to better characterize the sources of dietary exposure Specific foods of this geographic area (such as strawberries and cherries) and specific traditional dishes (like the gazpacho, a Spanish-style soup made from tomatoes and other vegetables and spices, served cold) were also included. The new restructuring is shown in (Table 1). The FFQ includes nine possible responses according to frequency of consumption and serving size of each food. The FFQ was administered twice during pregnancy, during the first and the third trimesters of pregnancy.

The 24-h dietary recall was chosen as the reference method to validate the FFQ. Pregnant women were asked to complete their dietary intake (all beverages and foods consumed in the past 24 h) on three non-consecutive days, including two weekdays and one weekend day. FFQs were administered by the same interviewer to reduce bias and to improve the response rate and accuracy of the data obtained. The i-Diet software (GSN, Spain) was used to estimate energy and daily nutrients intake [29]. This software was originally developed to generate healthy diets by dieticians and nutritionists and contains a large database of Spanish foods that allows estimating the intake of more than 50 nutrients. It has been widely used over the last years in epidemiological and dietary intervention studies to estimate nutrient intakes in study populations.

### 2.3. Data Analysis

Medians, means and standard deviations (SD) for total nutrient intakes were calculated for the two FFQs and three 24-h dietary recalls administered to pregnant women. A paired-sample sign test was applied to compare the medians of total nutrient intakes in the two study periods (first and third trimesters of pregnancy). The residual method of Willett was used to adjust macro and micronutrients for energy intake [19]. The reproducibility of the FFQ was assessed using two different statistical approaches: The Spearman correlation coefficient and a classification of the nutrient intakes divided into quintiles. Correlations were calculated depending on every nutrient before and after adjusting for energy; additionally, nutrient intakes were divided into quintiles and the percentage of correctly classified subjects into the same or adjacent quintiles was calculated. Both statistical techniques were also used to evaluate the validity of the FFQ by comparing the total nutrient intakes obtained by FFQ and 24-h dietary recalls. The limit of agreement (LoA) technique was also used in validation. LoA technique or Bland-Altman method is based on a graphical technique, whose limits of agreement were established as ±1.96 SD of the mean difference between the total nutrient intakes obtained in FFQs and 24-h dietary recalls [27].

Finally, the mean nutrient intake of the two FFQ results was compared with the dietary reference values for each nutrient recommended by the European Food Safety Authority (EFSA) [30] for pregnant women by calculating the percentage of the relative difference from the recommended values. Furthermore, these average values were compared with other studies in pregnant women. All analyses were performed with the SPSS 22.0 statistics package. The level of significance was established at *p* < 0.05.

## 3. Results

### 3.1. Characteristics of Participant Pregnant Women

Table 2 presents the main characteristics of the 690 pregnant women: the mean age of women was 31 with a standard deviation of 4.9 years old, 32.9% had overweight or obese before pregnancy, 84.4% were Spanish, 26.8% had university studies, 94.5% of women lived with their couples, 61.3% were primiparous, 5.5% were diagnosed of hypertension and/or diabetes, 12.5% smoked during the entire pregnancy and 4.3% only during first trimester, 6.2% reported alcohol consumption during the first trimester and 1.9% during the third trimester of pregnancy. Supplement consumption of folic acid, vitamin B complex and Vitamin complex was reported by 92%, 0.7% and 3.8% of women, at first trimester and by 95.8%, 0.9% and 1.7% at the third trimester, respectively.

### 3.2. Reproducibility

Table 3 shows the median daily intake of nutrients (assessed by two FFQs) for 690 pregnant women. Intake of all nutrients was slightly lower in the third trimester of pregnancy (*p* < 0.01 in sign test). The Spearman correlation coefficients for nutrients estimated by the two FFQs are also presented in Table 3. Significant correlations were observed for all estimated nutrients. Correlations coefficients ranged from 0.509 to 0.297 for vitamin B6 and E, respectively. When the analysis was based on energy-adjusted nutrient intakes, higherand statistically significant Spearman correlation coefficients were found for most nutrients (Table 3). According to quintile classification for nutrient intake, the percentage of subjects in the same or adjacent quintile, estimated by the two FFQs, ranged from 72.6% to 60.5% for alcohol and molybdenum, respectively (Table 3).

### 3.3. Validity

Among the 690 participants, a subsample of 43 was selected for the validation analysis (Table 4). These pregnant women filled in three 24-h dietary recalls in addition to the FFQs. Median daily energy and nutrient intakes based on FFQ and 24-h dietary recalls are presented in Table 4. Similar values (paired-sample sign test) were observed between the different methods for vitamins (except B6 and Biotin), minerals (except sodium, potassium, magnesium, iron, molybdenum, chromium and cobalt) and others (except for protein, saturated fat, cholesterol and energy). Those showing significant differences had slightly lower values in the FFQs. The Spearman correlation coefficients of nutrients intake adjusted for energy were calculated and only 13 of the 31 estimated values were statistically significant. These correlation coefficients ranged from 0.465 for cobalt to 0.305 for cholesterol.

**Figure 1:**
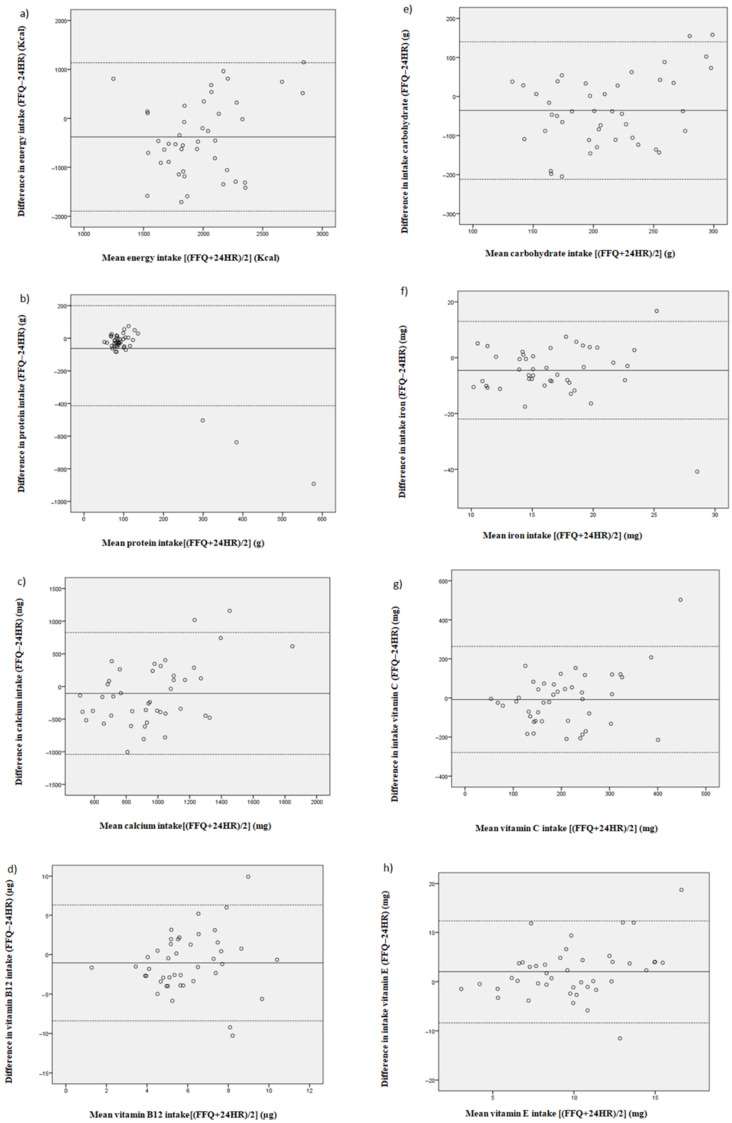
Bland–Altman plot. Bland–Altman plot between food frequency questionnaire (FFQ) and 24-h dietary recalls methods for measuring daily (**a**) energy, (**b**) protein, (**c**) calcium, (**d**) vitamin B12, (**e**) carbohydrate, (**f**) iron, (**g**) vitamin C and (**h**) vitamin E intake. Solid lines represent mean differences between the two methods. Dashed lines represent the limits of agreement corresponding to ±1.96 SD.

On the other hand, the percentage of subjects classified in the same or adjacent nutrient intakes’ quintile varied from 44.1% for total energy to 74.4% for cobalt. As commented before, the correlation coefficient is not a measure of agreement but a measure of association and can be partly influenced by the sample size. For this reason, we also applied the Bland-Altman method and calculated the percentage of subjects into the limits of agreement (Table 4). This varied from 83.7% for alcohol to 97.6% for total energy, vitamin C and Niacin. Figure 1 illustrates some graphics of the Bland-Altman method.

### 3.4. Nutrients Intake in Pregnant Women

**Figure 2:**
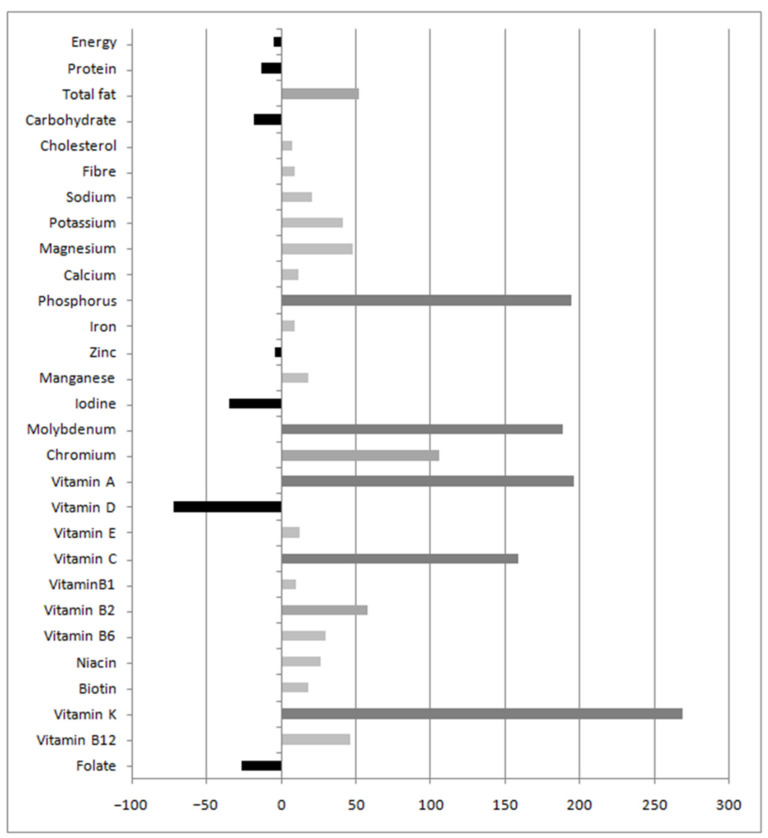
Percentage of the relative difference between the mean of nutrient intakes during pregnancy and the recommended values. Percentage of the relative difference from the mean of nutrient intakes during pregnancy of FFQs compared with dietary reference values established for each nutrient by the European Food Safety Authority (EFSA).

Figure 2 illustrates the comparison of the nutrients intake with the dietary reference values for each nutrient for pregnant women [30]. Percentages of the relative difference from the values were near or above the dietary reference values. Some micronutrients doubled or tripled the recommendations, such as vitamin K (268.5%), vitamin C (158.7%), vitamin A (195.6%), molybdenum (188.7%) and phosphorus (194.1%). Conversely, other nutrients such as iodine (−35.4%), vitamin D (−72%) and folate (−27%) were notably below the recommended values.

Table 5 shows the comparison of our results with seven other studies carried out on pregnant women. Although some nutrients show a wide difference, our results are within the ranges shown by other studies. Nutrient intakes were similar and comparable with other studies, especially of Spanish populations.

## 4. Discussion

### 4.1. Validation and Reproducibility

The results of this study demonstrate the validity and reproducibility of a 141-item modified FFQ based on the one previously validated by Vioque et al. (2013). The original FFQ was modified to meet the requirements of the target population of the GENEIDA birth cohort. Despite the use of biomarkers represents the method of preference to validate the FFQ, it could have some limitations for pregnant women because of the use of food supplements. Thus, we decided to validate the FFQ with 24-h dietary recalls.

Overall, the modified FFQ has a good validity and a huge reproducibility for all nutrient intakes during pregnancy. Regarding the external validity of this study, it should be noted that our study population has a medium-low socioeconomic level, with a 15% immigrant population, mainly of Latin American origin; however, the lifestyle of 85% of the population is similar to that of other pregnant women from other Spanish regions. Hence, the questionnaire could be used in other studies carried out in Spain, and more specifically in Andalusian population, showing similar characteristics to the pregnant women participating in this study.

In our study, the average correlation coefficients for reproducibility between the first and second administration of the FFQ was 0.41 for the 32 nutrients intake considered. This value is lower than that obtained in the INMA-Valencia study in Spain [18], where the average of the correlations coefficient was 0.51. The difference may be due to the use of different correlation coefficients. We have used the Spearman’s correlation coefficient as the variables analyzed were non-normally distributed, while the INMA-Valencia study used the Pearson’s correlation coefficient. On the other hand, our study compared the results of application of the FFQ at two different time-points of the pregnancy and found different results, as a clear decrease in the nutrient intake was observed in the third trimester of pregnancy relative to the first trimester. However, the INMA-Valencia study showed similar results in the two applications of the FFQ, which can justify their higher correlations. When the means of the classification percentages in the same or adjacent quintile were compared, both studies found similar results, with 71% for INMA-Valencia study and 66% for our study.

The correlation coefficients for most of the nutrients were similar to other validation studies of FFQ in pregnant women [23,25]. In the current study, although significant correlation coefficients were observed for several nutrients, no significant correlations were found for proteins, saturated fat, sodium, iron, phosphorus, vitamin B2 or biotin, among others. The correlation coefficients obtained for the reproducibility test were better than those found for the validation. This is because the reproducibility assessment measures the correlation between the same test performed at two different times (first and third trimester of pregnancy) while the validation assessment measures the correlation between two different tests used to measure the same event. Hence, correlation studies are not recommended to evaluate the comparability between methods [33]. An alternative analysis was proposed in 1983, based on the quantification of the agreement between two quantitative measurements, which is the current widely used method in nutritional epidemiology. The Bland-Altman method has been used to evaluate the agreement between the two methods in several validation studies conducted for pregnant women [22,23]. Therefore, the Bland-Altman analysis was used to evaluate the agreement between questionnaires regarding validation. Figure 1 shows points distribution within the LoA of some nutrients, both in the validation and in the reproducibility assays. When results are shown in percentages (Table 4), all values are above 90%, except for alcohol (83.7%), from which it can be concluded that both methods are comparable.

Another appropriate way to access the agreement between two methods is, again, the percentage of agreement by quintile. According to classification into quintiles of nutrient intakes as estimated by the FFQ and the 24-h dietary recalls, between 44% (energy) and 74% (cobalt) of women were classified in the same or adjacent quintile with an average of 60%. The results obtained were comparable to those reported in other studies conducted with pregnant women as well [22,25].

### 4.2. Nutrients Intake in Pregnant Women

In the current study, pregnant women were not consuming the amounts recommended for the gestational period (Figure 2). In this case, deficiency or excess of nutrients was calculated using dietary reference values established for each nutrient by EFSA [30]. Nutrients such as total fat, vitamin B2 and chromium were above 50% recommended daily allowance. Specifically, the intake of some nutrients such as sodium, total fat, saturated fat or cholesterol were above the reference values recommended. This should be closely monitored, because a high intake of these nutrients could pose a risk to the health of pregnant women and the fetus due to e.g., an increased risk of hypertension and cardiovascular complications [34]. Despite some micronutrients, such as phosphorus, vitamin K, vitamin A, vitamin C and molybdenum were above 150% of the recommendations, none of them were close to toxicity values considered harmful to the health of pregnant women. The highest deficiencies in the intake of micronutrients were found for iodine, vitamin D, and folate. These nutrients are essential during pregnancy and fetal development [11] and usual supplementation helps improve the inadequate intake. For example, in our study all participants received folate supplementation during pregnancy (400 micrograms/day), as recommended by public health agencies, which contributed to achieve the recommended folate levels despite their dietary deficits. Our study strengthens the idea that supplementation during pregnancy is crucial and that minor modification of the diet can improve all the deficits found. e.g., the use of iodized salt instead of common salt.

The comparison of these results with those of other studies (Table 5) showed that the nutritional status of pregnant women in South-East of Spain are within the range reported by other studies, especially if they are from European populations [12,15,22,23,25,26,31,32]. For example, percentages of inadequacy for vitamin D and iodine are similar to those observed by other epidemiological studies conducted in Spanish population [12,15,31]. The population from Southern Spain is characterized by a high dietary diversity, so it is not unusual to obtain similar values to other studies conducted in different Spanish population [15,31]. Specifically, our study shows similar results for all macronutrients and some micronutrients such as calcium or vitamin E to those observed for pregnant women in Valencia, Spain [15]. Intakes of other vitamins, such as vitamin A or vitamin C and group B vitamins, were comparable to another study performed in Spanish pregnant women [31]. However, for other micronutrients such as B vitamins and some minerals such as phosphorus and iodide, the results are close to those found in European studies [23,26]. Conversely, results of studies conducted in Asian populations show more disparate values, either higher or lower, than those described in our study [22,25,32].

These variations cannot only be due to differences in servings and type of food ingested but also to the food composition tables used. It should be noted that the present study collects values of the intake of molybdenum, chromium, cobalt or vitamin K, that are rarely described in studies of pregnant women, so it is difficult to obtain references to compare the intake of these nutrients. Although this study has some limitations, such as not using biochemical parameters for validation and the low number of subjects used, the results obtained are comparable with those of other populations with similar characteristics. Furthermore, the comparisons made with other studies allows us to infer that our results are reasonable and therefore the FFQ developed is a useful tool.

## 5. Conclusions

Overall, this study shows a good validity and reproducibility for measuring most of nutrient intakes. Thus, the present FFQ becomes a valid tool to collect dietary data for South-East Spanish pregnant women. The results of this study suggest that the diet of pregnant women living in this area ensures a good intake of nutrients and, together with vitamin supplementation, can meet the necessary nutritional requirements to guarantee the health of the fetus. In addition, we have included data for some nutrients like vitamin K, chromium, cobalt, or molybdenum, which are not commonly reported in studies on pregnant women. Finally, the population of this study is very similar in terms of nutrients intake to other Spanish populations of pregnant women.

## Figures and Tables

**Table 1:** Structure of the FFQ separated by groups, subgroups and number of items.

Food Groups of FFQ	Subgroups of Food	Items for Groups
Dairy products	Milk	4
	Cheese	2
	Dairy Derivatives	5
Meat, fish and eggs	Meat	6
	Meat Derived	7
	Fish	9
	Other	4
Vegetables	Vegetables	14
	Spices	2
	Tubers	3
Legums	Pulses	4
Fruits	Fruits	11
	Derived of Fruits	3
	Nuts	2
Breads and cereals	Bread and Derived	3
	Cereals	2
	Other	3
Oils and fats	Oil	7
	Other fats	3
Bakery and Pastry	Biscuits	6
	Cakes	6
	Chocolate	2
	Other	2
Drinks	Alcoholic drinks	9
	Other drinks	10
Mixed	Fried	3
	Sauces	4
	Other	5

**Table 2:** Characteristics of participant pregnant women of the GENEIDA study (*n* = 690).

Variables	Mean ± SD/n (%)
Woman’s age at assessment (years) (mean ± SD)	31.1 ± 4.9
Weight before pregnancy (kg) (mean ± SD)	64.8 ± 13.0
Weight at 32 week of gestation (kg) (mean ± SD)	76.1 ± 12.7
Weight gained through week 32	11.2 ± 5.4
Classified BMI pre-pregnancy ^1^	
Underweight (<18.5)	26 (3.8)
Normal (18.5–24.99)	432 (62.6)
Overweight (25.00–29.99)	162 (23.5)
Obesity (≥30)	65 (9.4)
Classified BMI ^1^	
Underweight (<18.5)	1 (0.1)
Normal (18.5–24.99)	135 (19.6)
Overweight (25.00–29.99)	347 (50.3)
Obesity (≥30)	202 (29.3)
Birth Country ^1^	
Spain	585 (84.4)
Other	105 (15.2)
Education level ^1^	
Primary or lower studies	45 (6.5)
Secondary studies	448 (64.9)
Higher studies	185 (26.8)
Family income (Euros/month) ^1^	
<500	22 (3.2)
500–1000	85 (12.3)
1001–2000	308 (44.6)
2001–3000	212 (30.7)
3001–5000	51 (7.4)
>5000	8 (1.1)
Status marital	
Married/Couple	652 (94.5)
Alone	38 (5.5)
Medical history	
Nothing	652 (94.5)
Diabetes	18 (2.6)
Hypertension	18 (2.6)
Diabetes and Hypertension	2 (0.3)
Parity	
≥1	267 (38.7)
0	423 (61.3)
Smoking	
No	574 (83.2)
1st trimester	30 (4.3)
All pregnancy	86 (12.5)
Alcohol consumption 1st trimester (gr/day) (mean ± SD)	4.5 (6.2)
Alcohol consumption 3st trimester (gr/day) (mean ± SD)	0.6 (1.9)
Supplements consumption 1st trimester ^2^	
Vitamin B complex	5 (0.7)
Vitamin complex	26 (3.8)
Folic acid	635 (92.0)
Nothing	40 (5.8)
Supplements consumption 3st trimester ^2^	
Vitamin B complex	6 (0.9)
Vitamin complex	12 (1.7)
Folic acid	661 (95.8)
Nothing	17 (2.5)

^1^ In these variables there are some missing data. ^2^ In these variables the categories are not exclusive, that is, an individual can present multiple categories.

**Table 3:** Mean daily energy and nutrient intakes based on food-frequency questionnaires, FFQ 1 and FFQ 2 (*n* = 690).

	FFQ1			FFQ2			Correlation Coefficient Unadjusted ^a^	Correlation Coefficient Adjusted ^b^	Agreement by Quintile (%) ^c^
	Mean	SD	Median	Mean	SD	Median			
Energy (kcal)	2401	742.7	2320	2052	671.3	1976 *	0.448 *		68.70
Protein (g)	103.2	29.51	100.1	89.94	28.67	86.23 *	0.408 *	0.411 *	65.80
Total fat (g)	109.2	39.43	102.2	94.02	36.87	87.36 *	0.407 *	0.434 *	64.49
Carbohydrate (g)	267.4	99.75	253.4	229.9	84.24	215.6 *	0.432 *	0.433 *	67.54
Saturated fat (g)	24.70	9.08	23.15	20.57	8.11	19.21 *	0.405 *	0.405 *	63.77
Cholesterol (mg)	347.9	126.1	327.1	291.7	111	273.5 *	0.415 *	0.464 *	64.49
Fiber (g)	34.48	13.66	32.31	30.61	13.24	29.17 *	0.416 *	0.443 *	67.83
Alcohol (g)	2.07	3.39	0.90	0.25	0.76	0.00 *	0.366 *	0.366 *	72.61
Sodium (mg)	2637	997	2494	2177	871.1	2037 *	0.366 *	0.381 *	61.59
Potassium (mg)	5175	1781	4934	4704	1768	4479 *	0.384 *	0.406 *	65.80
Calcium (mg)	1175	430.8	1128	1048	398.5	1005 *	0.387 *	0.405 *	66.67
Magnesium (mg)	454.5	152.1	439.9	431.9	165.7	410.9 *	0.359 *	0.381 *	65.65
Phosphorus (mg)	1704	511	1637	1531	504.1	1475 *	0.430 *	0.427 *	67.83
Iron (mg)	18.69	5.98	18.01	16.12	5.58	15.52 *	0.426 *	0.432 *	67.39
Zinc (mg)	13.24	4.02	12.88	11.32	3.80	10.84 *	0.437 *	0.459 *	66.96
Manganese (µg)	3750	1309	3643	3291	1296	3132 *	0.395 *	0.407 *	63.04
Iodine (µg)	139.2	58.22	133.3	119	52.38	111.8 *	0.443 *	0.432 *	66.52
Molybdenum (µg)	195.8	95.61	177.5	179.5	90.76	168.5 *	0.373 *	0.401 *	60.58
Chromium (µg)	65.64	25.63	63.24	57.79	24.76	55.14 *	0.372 *	0.372 *	66.96
Cobalt (µg)	26.40	12.91	24.43	22.83	12.14	20.28 *	0.445 *	0.447 *	67.54
Vitamin A (µg)	2234	1216	1870	1904	1112	1624 *	0.418 *	0.396 *	61.59
Vitamin E (mg)	13.11	5.04	12.27	11.40	4.69	10.71 *	0.297 *	0.301 *	60.87
Vitamin D (µg)	4.65	2.46	4.11	3.74	2.04	3.29 *	0.435 *	0.458 *	63.04
Vitamin C (mg)	299.1	149.4	272.7	244.2	125.6	218.7 *	0.344 *	0.337 *	60.58
Vitamin B1 (mg)	1.76	0.56	1.70	1.51	0.53	1.46 *	0.446 *	0.450 *	68.70
Vitamin B2 (mg)	2.36	0.78	2.25	2.05	0.71	1.96 *	0.449 *	0.449 *	68.70
Vitamin B6 (mg)	2.63	0.85	2.54	2.28	0.83	2.19 *	0.509 *	0.502 *	72.03
Niacin (mg)	26.31	8.69	25.29	24.08	9.28	23.01 *	0.337 *	0.375 *	62.90
Biotin (µg)	48.78	17.40	46.73	45.05	18.16	42.58 *	0.469 *	0.479 *	69.28
Vitamin K (µg)	371.5	161.7	352.7	291.8	137.8	265.9 *	0.419 *	0.402 *	66.67
Vitamin B12 (µg)	7.00	2.82	6.59	6.10	2.88	5.53 *	0.385 *	0.387 *	60.87
Folate (µg)	462.8	167.4	447.7	412.3	165.5	393.1 *	0.472 *	0.486 *	70.00

^a^ Spearman Correlation coefficient using unadjusted nutrient intakes. ^b^ Spearman Correlation coefficients when adjusting for total energy intake. ^c^ Percentage of the subjects classified in the same or adjacent nutrient intakes’ quintile. * Correlation significant at *p* < 0.01.

**Table 4:** Mean daily energy and nutrient intakes based on food-frequency questionnaire (FFQ) and 24-h dietary recalls (n = 43).

	FFQ			24-h Dietary Recalls			Correlation Coefficient ^b^	Agreement by Quintile (%) ^c^	Agreement by LoA (%) ^d^
	Mean	SD	Median	Mean	SD	Median ^a^			
Energy (kcal)	1745	530	1646	2165	437.6	2133 *		44.19	97.67
Protein (g)	80.69	30.37	73.7	97.07	21.51	101.6 *	0.217	58.14	93.02
Total fat (g)	78.17	23.50	78.94	99.97	25.32	100.1	0.113	60.47	95.35
Carbohydrate (g)	192.7	74.89	182.2	228.4	51.28	234.1	0.300	67.44	95.35
Saturated fat (g)	16.12	4.69	17.25	28.15	9.01	28.77 **	0.176	53.49	95.35
Cholesterol (mg)	227.8	63.02	236.1	228.2	70.08	439.2 **	0.305 *	55.81	93.02
Fiber (g)	26.64	13.19	25.36	24.04	6.99	24.68	0.358 *	62.79	93.02
Alcohol (g)	0.19	0.56	0.00	0.54	1.56	0.00	0.341 *	60.47	83.72
Sodium (mg)	1849	612.8	1857	2500	836.6	2463 **	0.130	51.16	93.02
Potassium (mg)	3742	1489	3424	4699	1304	4450 *	0.332 *	67.44	95.35
Calcium (mg)	850.2	362.3	818.7	1014	267.9	1028	0.397 **	62.79	95.35
Magnesium (mg)	327.5	115.7	325.5	416.5	103	417.8 *	0.378 *	62.79	95.35
Phosphorus (mg)	1378	556.6	1287	1502	323.9	1503	0.299	62.79	93.02
Iron (mg)	14.35	5.82	13.41	18.19	4.37	17.92 *	0.113	60.47	95.35
Zinc (mg)	10.49	4.02	9.70	10.79	2.95	11.09	0.433 *	53.49	97.67
Manganese (µg)	3150	1512	2721	3004	796.2	2935	0.228	62.79	93.02
Iodine (µg)	107.7	47.8	105.4	99.26	40.17	91.70	0.257	62.79	95.35
Molybdenum (µg)	140.9	56.92	138.2	222.8	99.18	202.3 **	0.349 *	65.12	93.02
Chromium (µg)	54.99	21.44	53.32	70.11	18.73	71.42 **	0.114	58.14	95.35
Cobalt (µg)	18.36	9.79	16.90	17.92	7.46	34.58 **	0.465 **	74.42	93.02
Vitamin A (µg)	1690	961.7	1657	1811	902.3	1701	0.197	65.12	93.02
Vitamin E (mg)	10.52	4.20	9.93	8.85	3.40	8.34	0.332 *	60.47	90.70
Vitamin D (µg)	3.01	1.47	2.90	3.58	2.28	3.74	0.271	55.81	95.35
Vitamin C (mg)	190.1	105	183.6	202.4	80.21	194.6	0.398 **	60.47	97.67
VitaminB1 (mg)	1.35	0.46	1.34	1.56	0.38	1.51	0.337 *	58.14	93.02
Vitamin B2 (mg)	1.89	0.79	1.80	1.95	0.47	2.03	−0.130	51.16	97.67
Vitamin B6 (mg)	2.12	0.87	1.90	2.36	0.61	2.42 *	0.391 **	67.44	95.35
Niacin (mg)	19.94	7.39	18.86	22.89	7.44	22.15	0.173	60.47	97.67
Biotin (µg)	35.30	12.81	35.35	47.89	14.36	49.04 **	−0.055	46.51	95.35
Vitamin K (µg)	283.5	142.1	240.1	313.8	179.8	305.1	0.283	55.81	95.35
Vitamin B12 (µg)	5.27	2.45	4.82	6.04	1.74	6.38	0.268	60.47	93.02
Folate (µg)	363.9	164.9	328.4	323.2	99.06	320.8	0.298	62.79	90.70

^a^ Paired-sample sign test. ^b^ Correlation coefficients of Spearman were adjusted for total energy intake. ^c^ Percentage of the subjects classified in the same or adjacent nutrient intakes’ quintile. ^d^ Overall proportion of agreement limits between both questionnaires. Corresponding to Bland–Altman plots. * Significant at *p* < 0.05 level; ** Significant at *p* < 0.01 level.

**Table 5:** Comparison of the daily average intake of energy and nutrients based on food frequency questionnaires (FFQ1 and FFQ2) with results obtained by other studies.

Nutrients	Our Study	1	2	3	4	5	6	7
Energy (kcal)	2226	2304		1923	2221	2021	1754	1744
Protein (g)	96.6	102		70	86	78.9	56	59
Total fat (g)	101.6	99		85.7	75	53.9	51	56
Carbohydrate (g)	248.6	261		228.3	300	306.4	263	242
Saturated fat (g)	22.6	31		32.9				18
Cholesterol (mg)	319.8	340		223.8			347	238
Fiber (g)	32.5	24		33.7	29	7	15	11
Alcohol (g)	1.1		3	0.1				
Sodium (mg)	2407	3411		2417		2586	481	3013
Potassium (mg)	4939			2532	3800	1647	2481	2454
Calcium (mg)	1112	1289		715.2	930	830.6	608	519
Magnesium (mg)	443.2	387		235.2	380		297	230
Phosphorus (mg)	1617			1153	1600	967.7	941	945
Iron (mg)	17.4	21		11.2	13	20.3	19	6.9
Zinc (mg)	12.3	28		7.8	11		9.5	7.2
Manganese (µg)	3520			1800				2500
Iodine (µg)	129.1	222		79.2	120		15.4	437
Molybdenum (µg)	187.7							
Chromium (µg)	61.7							
Cobalt (µg)	24.6							
Vitamin A (µg)	2069		1900	1500		987.7	872	909
Vitamin E (mg)	12.3	11.4	8	4.3	10		8	
Vitamin D (µg)	4.2	3.1	6	2.7	3.3			4.2
Vitamin C (mg)	271.6	144	253	73.9	160	127.2	164	111
VitaminB1 (mg)	1.6		2	1.5	1.5	1.6		0.9
Vitamin B2 (mg)	2.2		2	1.3	1.8	2		1.1
Vitamin B6 (mg)	2.5	2.1	2	2	1.6			1.2
Niacin (mg)	25.2		34	18.5	31	15.9	11	13.3
Biotin (µg)	46.9			18.2				
Vitamin K(µg)	331.6							219
Vitamin B12 (µg)	6.5	9.9	6	3.5				4.2
Folate (µg)	437.5	305	400	229.2	280		133	284

1 Vioque et al., 2013 [15]; 2 Salcedo-Bellido et al., 2017 [31]; 3 Mouratidou et al., 2006 [23]; 4 Brantsaeter et al., 2007 [26]; 5 Loy et al., 2011 [32]; 6 Zhang et al., 2015 [22]; 7 Ogawa et al., 2017 [25].
